# Anxiety in heart failure patients and its association with socio-demographic and clinical characteristics: a cross-sectional study

**DOI:** 10.1097/j.pbj.0000000000000177

**Published:** 2022-09-09

**Authors:** Filipa M. D. Costa, Sónia P. V. Martins, Emilia C. T. D. Moreira, José C. M. S. Cardoso, Lia P. N. S. Fernandes

**Affiliations:** a Faculty of Medicine - University of Porto, Porto, Portugal,; b Department of Clinical Neurosciences and Menta Health, Faculty of Medicine - University of Porto, Porto, Portugal,; c Center for Health Technology and Services Research (CINTESIS), Faculty of Medicine - University of Porto, Porto, Portugal,; d Department of Medicine, Faculty of Medicine - University of Porto, Porto, Portugal,; e Department of Cardiology, Centro Hospitalar Universitário S. João, Porto, Portugal,; f Psychiatry Service, Centro Hospitalar Universitário S. João, Porto, Portugal.

**Keywords:** anxiety, GAD-7, heart failure, mental health, New York Heart Association functional classes, psychiatric disorder

## Abstract

Anxiety is one of the most frequent psychiatric disorders in heart failure (HF) patients. However, it is often neglected in clinical practice and studies about the particular relationship with the New York Heart Association (NYHA) classes for HF are scarce. In this context, this study aims to analyze the presence of anxiety symptoms in HF outpatients and also its association with sociodemographic and clinical characteristics of these patients.

**Methods:**

This cross-sectional study is part of the longitudinal Deus Ex-Machina project (NORTE-01-0145-FEDER-000026). HF patients were recruited at an outpatient clinic at a University Hospital. Patients with inability to communicate, severe visual/hearing impairment, or NYHA class IV were excluded. Sociodemographic data and NYHA class were registered. Anxiety was assessed with the 7-item Generalized Anxiety Disorders Scale (with a score ≥10 clinically relevant anxiety). Patients with and without anxiety were compared regarding socio-demographic and clinical variables.

**Results:**

The sample (n = 136) had a median age of 59years (Q_1_: 49; Q_3_: 68), 66.2% were male and 31.6% presented clinically relevant anxiety. A higher percentage of HF patients with anxiety had psychiatric disorders (58.1% vs 26.9%; *P* = .001), psychotropic medication (62.8% vs 30.1%; *P* = .001), and depression (60.5% vs 9.7%; *P*< .001). No significant differences were found regarding the remaining variables, including NYHA classes.

**Conclusions:**

A substantial proportion of HF patients present clinically relevant anxiety, particularly those with psychiatric history, depressive symptoms, or under psychotropic medication. Therefore, integrating routine screening and treatment of this comorbidity in clinical practice is of utmost importance. Further studies are needed to clarify the association of anxiety with HF.

## Introduction

Heart failure (HF) is a global and growing,^1^ public health problem,^2^ affecting more than 64 million individuals worldwide.^3^ Due to the advances in treatment and prevention of cardiovascular diseases and increased longevity in industrialized countries, it is expected that the number of patients diagnosed with HF will increase in the next years.^4−6^

HF is the leading cause of mortality in older patients,^2^ with approximately 50% of the patients dying within 5 years of diagnosis,^7^ and also of morbidity, driving to a significant economic burden, and a negative impact on patients, families, and society.^3,8^

According to the European Society of Cardiology, HF is “a clinical syndrome characterized by typical symptoms (eg, breathlessness, ankle swelling, and fatigue) that may be accompanied by signs (eg, elevated jugular venous pressure, pulmonary crackles, and peripheral edema) caused by a structural and/or functional cardiac abnormality, resulting in a reduced cardiac output and/or elevated intra-cardiac pressures at rest or during stress”.^9^

According to left ventricular ejection fraction (LVEF), HF can be classified into HF with reduced ejection fraction (LVEF <40%); HF with mid-range ejection fraction (LVEF 40%–49%); HF with preserved ejection fraction (LVEF ≥ 50%).^9^

New York Heart Association (NYHA) functional classification builds on HF symptom severity to define 4 classes^10^: class I—no limitation of physical activity, class II—slight limitation of physical activity, class III—marked limitation of physical activity, and class IV—unable to carry on any physical activity without discomfort, symptoms at rest can be present.^9^

Among HF patients, psychiatric disorders are prevalent, namely depression and anxiety.^11−13^ Approximately 40% of HF patients experience major anxiety, and general anxiety levels are 60% higher than the ones observed in the general population.^12^

Anxiety is a negative affective state that results from a perception of threat,^12^ and preparation for possible, future negative events.^14^ It may be a normal response to the diagnosis of HF, but the presence of anxiety symptoms in patients with cardiac disease is not benign if it is extreme or persists.^12^

The investigation regarding the association between anxiety and HF outcomes, such as re-hospitalization and mortality is still sparse, and results are inconsistent.^2,12,15−17^ However, other HF outcomes such as quality of life and functional status at 1 year are proven to be adversely impacted by anxiety.^2,12^

Despite the high prevalence of anxiety in HF patients, this psychiatric disorder is often neglected in clinical practice and frequently underdiagnosed.^15,18,19^ The under recognition of anxiety may be partially explained by the overlap of psychiatric and cardiac symptoms, such as palpitations, chest pain, shortness of breath, among others.^20^ Therefore, this comorbidity is seldom treated,^15,18,19^ and, even when assessed and treated, follow-up is lacking and symptoms commonly persist.^21^

Studies about anxiety in HF patients are still scarce, particularly its relationship with some clinical characteristics, such as with the NYHA functional classes. Bearing this in mind, the present study aims to analyze the presence of anxiety symptoms in HF outpatients and also its association with socio-demographic and clinical characteristics of these patients.

## Methods

### Sample recruitment

This cross-sectional study is part of the longitudinal project: “Symbiotic technology for societal efficiency gains - Deus Ex Machina (DeM)” (NORTE-01-0145-FEDER-000026). HF patients were randomly recruited using the outpatient list of University Hospital HF clinic. Data collection was collected between September 2017 and September 2018. Inclusion criteria were age 18 years and over, clinical diagnosis of HF based on the European Society of Cardiology criteria.

NYHA class IV patients, or those unable to communicate or with severe visual/hearing impairment, were excluded.

This study was conducted according to the Strengthening the Reporting of Observational Studies in Epidemiology (STROBE) Statement: guidelines for reporting observational studies.^22^

### Ethical considerations

This study was approved on July 11, 2017 by the Ethics Committee for Health of the Centro Hospitalar Universitário de Säo João (CHUSJ), in Porto, with reference number: 57/17. Written informed consent of all study participants was obtained at enrolment in this study.

### Procedures

After written informed consent, the patients were assessed in a private room, with the research protocol, with an average duration of between 1 hour and an hour and a half. This protocol had a multidisciplinary approach, applying measurement instruments from Cardiology, Psychology, Psychiatry, and Nutrition fields. The Cardiology variables were collected by Cardiologists from the outpatient clinic, whilst the Psychology/Psychiatry and the Nutrition ones were collected by Psychologists and Nutritionists from the research team.

### Measures

Complete socio-demographic characteristics of patients and hospital-related data were obtained through a clinical interview and medical chart review. In the following, 2 assessment instruments of the research protocol will be described considering the objectives of this work:

#### 1. The Generalized Anxiety Disorders Scale

For assessing anxiety, the 7-item Generalized Anxiety Disorders Scale (GAD-7),^23^ validated for use in Portugal,^24^ was applied. This questionnaire is composed of 7 items which assesses the presence of anxious symptoms for the past 14 days. The answers are given in a scale from 0 to 3, respective to the frequency of the symptoms (0 = not at all, 1 = several days, 2 = more than half of the days, and 3 = nearly every day). The total score ranges from 0 to 21, where a higher score means more severe anxious symptomatology. In this study, the following anxiety severity categories recommended by Spitzer et al,^23^ were used: none/normal (0–4), mild (5–9), moderate (10–14), and severe (15–21). A final score ≥10 was considered to indicate presence of clinically relevant anxiety.

#### 2. Patients Health Questionnaire

The Portuguese validated version of the Patient Health Questionnaire-9 (PHQ-9),^25,26^ was used as depression measure. A total of 9 items constitutes this questionnaire, assessing the presence of depressive symptoms for the last 14 days. The answers are given in the same scale as of GAD-7’s, with the total ranging between 0 and 27. A higher score means more severe depressive symptomatology. Regarding depression severity categories, were followed the recommendations of Kroenke et al^25^: minimal (0–4), mild (5–9), moderate (10–14), moderately severe (15–19), and severe (20–27). A total score ≥10 was considered to indicate presence of clinically relevant depression.

### Statistical analysis

Statistical analyses were performed using SPSS version 26.0 for Windows (SPSS, Inc., Chicago, IL).

Non-parametric tests were conducted and patient sociodemo-graphic and clinical characteristics were presented as raw frequencies and percentages for categorical variables, and as median and range for continuous variables (since normality was not assumed). Normality was verified by distribution analysis and the Kolmogorov-Smirnov test.

For analysis of differences between the groups of patients with or without anxiety, the Mann-Whitney and Kruskal-Wallis tests were performed for continuous variables, and the Chi-Square test for the categorical variables. The level of statistical significance for all tests was defined by *P <* .050.

## Results

A total of 139 patients were recruited. During the data processing, 3 patients were excluded for incomplete data, resulting in a final sample of 136 patients.

The final sample had a median age of 59years (Q_1_: 49; Q_3_: 68), and the majority was male (66.2%), married (75.7%), and had a median education of 9years (Q_1_:4;Q_3_: 12). Most of the patients were retired (55.9%) and had a monthly family income between 1000€ and 3000€ (52.2%). Moreover, mostly lived with partner and/or son/daughter (85.3%) and had a caregiver (87.5%).

Detailed socio-demographic characteristics of the global sample of HF patients are presented in Table 1.

**Table 1:** Socio-demographic characteristics of the patients with and without anxiety.

	Overall	With anxiety	Without anxiety	
	(n = 136)		(n=43)		(n = 93)		
Characteristics	n	%	n	%	n	%	*P* value
Age, (years) median (Q_1_−Q_3_)	59 (49−68)	56 (46−70)	59 (52−67.5)				*P* = .490^*^
Gender							*P* = .123^†^
Female	46	33.8	19	44.2	27	29.0	
Male	90	66.2	24	55.8	66	71.0	
Marital status							*P* = .736^‡^
Married	103	75.7	32	74.4	71	76.3	
Single	10	7.4	2	4.7	8	8.6	
Divorced/separated	18	13.2	7	16.3	11	11.8	
Widowed	5	3.7	2	4.7	3	3.2	
Education (years)							*P*=.188^†^
0–4	47	34.6	14	32.6	33	35.5	
5–9	47	34.6	20	46.5	27	29.0	
10–12	30	22.1	6	14.0	24	25.8	
>12	12	8.8	3	7.0	9	9.7	
Professional situation							*P*=.783^‡^
Active worker	41	30.1	12	27.9	29	31.2	
Unemployed/sick-leave	14	10.3	6	14.0	8	8.6	
Other	5	3.7	2	4.7	3	3.2	
Retired	76	55.9	23	53.5	53	57.0	
Monthly family income							*P*=.088^‡^
<1000€	60	44.1	24	55.8	36	38.7	
≥1000€ and 3000€	71	52.2	19	44.2	52	55.9	
≥3000€	5	3.7	0	0.0	5	5.4	
Living situation							*P*=.797^‡^
Alone	11	8.1	3	7.0	8	8.6	
With partner and/or son/daughter	116	85.3	38	88.4	78	83.9	
Others	9	6.6	2	4.7	7	7.5	
Has a caregiver?							*P*=.626^†^
Yes	119	87.5	39	90.7	80	86.0	
No	17	12.5	4	9.3	13	14.0	

Q_1_c0200A;=first quartile, Q_3_=third quartile.

* Mann-Whitney test.

† Chi-Square Independent test.

‡ Chi-Square exact test.

Regarding the type of HF according to LVEF, 50%, 25%, and 25% of the patients were classified as HF with reduced ejection fraction, HF with mid-range ejection fraction, and HF with preserved ejection fraction, respectively. Regarding NYHA class, 36%, 49.3%, and 14.7% were at class I, II, and III, correspondingly (Table 2).

**Table 2:** Clinical characteristics of the patients with and without anxiety.

	Overall	With anxiety	Without anxiety	
	(n = 136)		(n=43)		(n = 93)		
Characteristics	n	%	n	%	n	%	*P* value
Type of HF according to LVEF							*P* = .379^*^
HFrEF	68	50.0	25	58.1	43	46.2	
HFmrEF	34	25.0	8	18.6	26	28.0	
HFpEF	34	25.0	10	23.3	24	25.8	
NYHA class							*P*=.206^*^
Class I	49	36.0	12	27.9	37	39.8	
Class II	67	49.3	26	60.5	41	44.1	
Class III	20	14.7	5	11.6	15	16.1	
History of psychiatric disorder							***P =*** .001^*^
Yes	50	36.8	25	58.1	25	26.9	
No	86	63.2	18	41.9	68	73.1	
Medication for anxiety/depression/sleep problems							***P =*** .001^*^
Yes	55	40.4	27	62.8	28	30.1	
No	81	59.6	16	37.2	65	69.9	
PHQ-9							***P<*** .001^*^
With depression	35	25.7	26	60.5	9	9.7	
Without depression	101	74.3	17	39.5	84	90.3	

The bold characters in the *P* value column refer to the significant *P* values (*P*< .05).

HF = heart failure, HFmrEF = heart failure with mid-range ejection fraction, HFpEF = heart failure with preserved ejection fraction, HFrEF = heart failure with reduced ejection fraction, LVEF = left ventricular ejection fraction, NYHA = New York Heart Association, PHQ-9 = Patient Health Questionnaire-9.

* Chi-Square independent test.

Regarding GAD-7 results, the median score of this questionnaire was 5 (Q_1_:2;Q_3_: 11), and 31.6% of the patients presented clinically relevant anxiety, according to the cut-off point of 10. Regarding the severity, 25% had moderate anxiety, 24.3% mild, and 6.6% revealed severe anxiety. In this study, 44.1% of patients had no anxiety.

**Figure 1:**
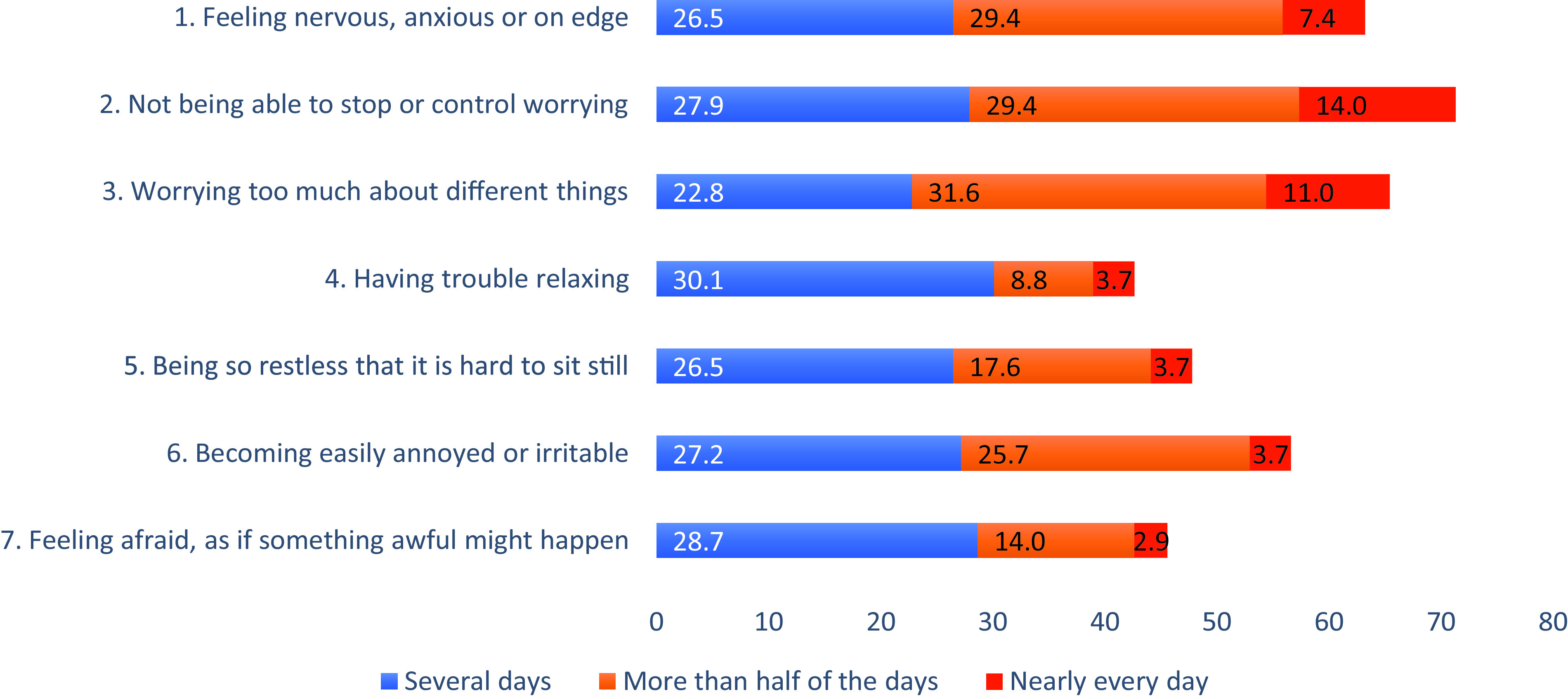
Frequency (%) of patients with anxiety symptoms according to GAD-7 items. GAD-7 = 7-item Generalized Anxiety Disorders Scale.

The 2 most frequent anxiety symptoms reported were “not being able to stop or control worrying” and “worrying too much about different things” with 71.3% and 65.4% of patients, respectively (Fig. 1).

Concerning depression, 25.7% had clinically relevant depression, according to PHQ-9 results. History of psychiatric disorder was reported by 36.8% of the patients and 40.4% take medication daily for anxiety/depression and/or sleep problems (Table 2).

Regarding the comparison between groups of patients with and without anxiety, no statistically significant differences were found concerning the socio-demographic characteristics (Table 1).

In relation to clinical characteristics, statistically significant differences were found between the 2 groups (with and without anxiety), in psychiatric history, with 58.1% of anxiety patients presenting pre-existing psychiatric disorder, while in the group without anxiety, this proportion was only of 26.9% (*P* = .001).

Also, significant differences were found for daily medication intake for anxiety/depression and/or sleep problems. In this study, a higher percentage of anxiety patients taking medication, compared to patients without anxiety (62.8%vs30.1%; *P* = .001).

Related to depression, assessed by PHQ-9, statistically significant differences were also found between the 2 groups, with 60.5% of anxiety patients presenting clinically relevant depression, while in the group without anxiety, this proportion was only of 9.7% (*P*< .001) (Table 2).

No differences were found for the remaining clinical variables evaluated in this study, including for the NYHA class.

## Discussion

The findings of this study revealed that a significant proportion (31.6%) of HF patients is suffering from clinically relevant anxiety, which is in accordance with a recent review that concluded that 30% of patients reported clinically significant levels of anxiety, assessed by questionnaires.^15^ However, the prevalence of anxiety found in the current study was higher than in a previous study (14.4%), that also assessed anxiety using the GAD-7,^20^ which may be explained by some methodological discrepancies between the studies.

The current study aimed to analyze the association between anxiety and socio-demographic, as well as clinical characteristics. In this context, comparing HF patients with and without anxiety, a higher proportion of patients with anxiety have psychiatric history and use of daily psychotropic medication. These findings are corroborated by a previous study, that revealed a significant association between anxiety and anti-depressive medication. This study in question also revealed a significant association between anxiety and the number of contacts with patients’ general practitioner within the previous year.^27^ This association may also be important to evaluate in the future.

Additionally, the presence of clinically relevant anxiety was found to be positively correlated with depressive symptomatology, corroborating former works carried out with HF patients,^5,27−29^ in other clinical populations, such as cancer patients,^30^ as well as in the general population.^31,32^

According to a review article about HF, the association between anxiety and depression persists across studies regardless of the assessment tool used to evaluate these symptoms.^33^ This correlation can be partially explained by the overlap in symptoms between the 2 disorders, particularly those of a physiological nature.^20^

In addition, in the present study, the prevalence of anxiety in HF patients was higher than the prevalence of depression (25.7%), as it was previously seen in a review reporting elevated rates of anxiety in over half of HF patients.^2^

Another goal of this study, was to analyze the association between anxiety and NYHA functional classes. However, no significant association was found between these 2 variables, which is in accordance with some previous studies.^5,27^ Similarly, no significant relation was verified between anxiety and LVEF, also in accordance with what was observed in other studies.^27,34^

Regarding socio-demographic characteristics, no statistically significant differences regarding the age, marital status, educational level, income level, and living situation were verified, which is in line with other researches.^5,27^

In this study, no significant association was found between anxiety and the sex of the patient. The same was observed in a previous study,^27^ in spite of other studies showing that anxiety was more frequent among women.^2,5^ These differences can be due to methodological differences, such as the inclusion of NYHA class IV patients,^5^ or the use of different anxiety measures.^2,5^

The present research emerges as a contribution to the knowledge about anxiety in these patients and its related factors. In addition, this work also provides a characterization of socio-demographic and clinical variables in HF outpatients. Another strength of this study was the use of a widely standardized instrument for anxiety assessment, which is also validated for use in Portugal.^23,24^

Despite the fact that no association was found in the present study, between anxiety and the NYHA classes, it was evident that a substantial proportion of HF patients is experiencing clinically relevant anxiety, which has considerable implications for their psychological well-being and health.^20^

The results of the present work should take into consideration some limitations. First, due to the cross-sectional study design, symptoms of anxiety were only measured during baseline assessment. Therefore, the timing of the evaluation may have influenced the scores. Second, this is a one-site study, with the selection of a sample recruited from an outpatient unit in the referred hospital, which may have limited the power to detect differences between groups. Third, the sample was not representative of HF patients in the general population, so future studies will be needed to confirm the generalizability of these findings. Lastly, the use of psychotropic medication can also be considered a confounding factor of the study, since it was not accounted for effect-adjustment.

Taking all of this in mind, further studies with larger samples and longitudinal design are required in order to build a better understanding of the relationship between anxiety and HF. Also, studies of anxiety as a covariate to other factors is also advised because it is frequently correlated with other factors, namely depression.

## Conclusion

In this study, a significant proportion of HF patients reported clinically relevant anxiety, particularly those with psychiatric history, psychotropic medication, and depressive symptoms. Therefore, the need of integrating routine screening and treatment of this comorbidity in clinical practice, is of utmost importance.

The deeper knowledge of the association between anxiety and HF will contribute to improve detection of this psychiatric condition, and to determine the best ways to manage HF patients, taking into consideration the adversities that these patients experience in their daily lives.
